# The Navigation Guide: Systematic Review for the Environmental Health Sciences

**DOI:** 10.1289/ehp.122-A283

**Published:** 2014-10-01

**Authors:** Julia R. Barrett

**Affiliations:** Julia R. Barrett, MS, ELS, a Madison, WI–based science writer and editor, has written for *EHP* since 1996. She is a member of the National Association of Science Writers and the Board of Editors in the Life Sciences.

For decades the field of clinical science has used systematic review methods to integrate research findings and present the results in a consistent and unbiased manner to support health-protective recommendations. An interdisciplinary team of clinical and environmental health scientists has now adopted principles of systematic review and applied them to the environmental health sciences in a framework called the Navigation Guide.^1^^,^^2^ In this issue of *EHP* a case study on the widespread environmental contaminant perfluorooctanoic acid (PFOA) puts the guide through its paces to test the process itself and to judge the strength and quality of evidence regarding the effects of PFOA on fetal growth.^3^^,^^4^^,^^5^

PFOA confers fire resistance and oil- and water-repellent properties to many manufactured products, including clothing, furniture, carpets, paints, and adhesives.^6^ After 60 years of use, the chemical is detectable throughout the environment.^6^ PFOA resists degradation, and the compound is ubiquitous in blood samples collected in nationally representative surveys in the United States and other developed countries.^6^^,^^7^

**Figure:**
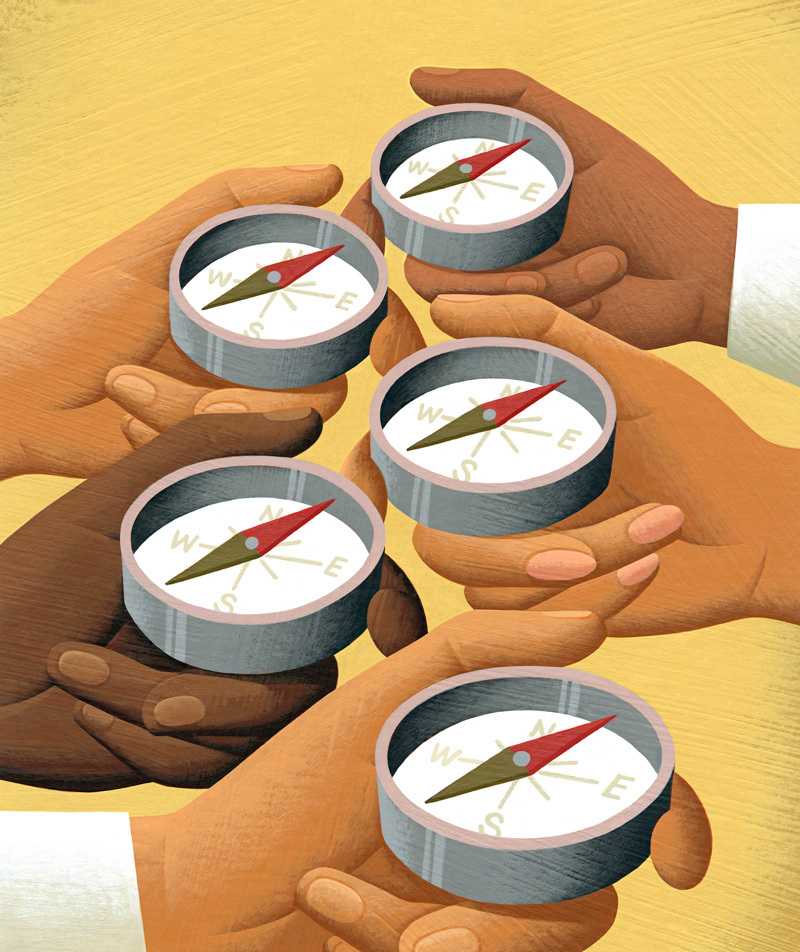
© Adam Niklewicz c/o the iSpot.com

Exposure has been associated with various adverse health outcomes in animals and humans, with one body of evidence centering on fetal growth.^7^ Impaired fetal growth is worrisome because it can have serious long-term implications.^1^ However, inconsistencies among study results hinder effective recommendations to protect health.

This is a problem not just for PFOA but also for any exposure suspected of causing harm. “There’s a boatload of new evidence coming out every month, but there has been no systematic way to evaluate it or deliberate about whether there’s sufficient evidence to declare whether something is toxic,” says Bruce Lanphear, a professor of health sciences at Simon Fraser University in Vancouver, British Columbia, who was not involved in developing the guide.

In developing the Navigation Guide, the team drew inspiration and guidance in particular from the Grading of Recommendations, Assessment, Development, and Evaluation (GRADE) approach, which is widely used by national and international medical societies and health organizations. This system rates the quality and strength of the evidence to allow for recommendations about specific clinical interventions. However, it is not directly transferable to the environmental health sciences, which have very different streams of evidence (e.g., a lack of randomized clinical trials, more emphasis on toxicology) and contexts for decision making (e.g., substances are typically assessed for their potential to harm rather than to heal).^1^ To address the integration of human and animal data, the authors also adapted elements of review processes used by the International Agency for Research on Cancer and the U.S. Environmental Protection Agency.^2^

The Navigation Guide comprises four steps: specify a research question, select the evidence, rate the quality and strength of the evidence, and determine a final recommendation to protect health. The guide uses a set of predefined systematic criteria for each step in the process of collecting and summarizing data. The goal is to minimize subjectivity and bias, and to maximize transparency and consistency in the hazard assessment step.

The team focused on PFOA as a case study.^5^ To answer the question “does developmental exposure to PFOA affect fetal growth in humans?” they conducted a systematic search of the literature. Ultimately, 18 epidemiological studies and 21 animal studies met the authors’ eligibility criteria for inclusion in the review. The studies were graded in terms of quality (high, moderate, or low) and strength (sufficient, limited, moderate, or lack evidence of toxicity). Results were then integrated according to clearly defined criteria, leading the coauthors to conclude that sufficient evidence exists to affirm that PFOA exposure decreases fetal growth in humans.

The PFOA case study highlighted points where the Navigation Guide was limited, such as research areas with gaps in knowledge. Identifying those gaps serves an important purpose. “The advantage of applying systematic and transparent approaches to evaluating the scientific literature is that all the information and decisions along the way are documented and justified with an explanation,” says coauthor Juleen Lam, an assistant scientist at the Johns Hopkins Bloomberg School of Public Health. “It will become apparent if there is not enough information to come to a strong conclusion, or if there is missing information or other research gaps.” Once research gaps are identified, action can be taken to fill them.

The researchers also identified a need for more precise definitions within the Navigation Guide, which would enhance the ability of scientists to reach strength-of-evidence conclusions and also help communicate findings to broader audiences such as policy makers and the general public. The scope of the guide needs to be expanded to capture a wealth of data from *in vitro* studies and other toxicological investigations.^5^

Nevertheless, the Navigation Guide is a welcome tool in environmental health. “I think that people have been pushing scientists to be very systematic and transparent in conveying how we arrive at decisions about whether a particular environmental hazard causes health risks,” says Matthew Strickland, an associate professor of environmental health at Emory University, who was not involved in the study. “It’s interdisciplinary in nature, and it’s challenging to come up with one system that everyone likes,” Strickland says. “But that’s science—we should expect that, and we should welcome that.”

The Navigation Guide exists alongside other evidence-integration methods currently being developed by the National Toxicology Program and the U.S. Environmental Protection Agency for similar purposes of reaching strength-of-evidence conclusions.^8^^,^^9^ This multiplicity is valuable, says Tracey Woodruff, a professor in the Department of Obstetrics, Gynecology, and Reproductive Sciences at the University of California, San Francisco, and senior author of the case study. “Having these different groups apply systematic and transparent methods of research integration to environmental chemicals independently helps strengthen our understanding of the issues that need to be investigated further, what kind of methodological aspects worked well, and which areas we need to improve,” she says.
